# An Overview of Anthropogenic Actions as Drivers for Emerging and Re-Emerging Zoonotic Diseases

**DOI:** 10.3390/pathogens11111376

**Published:** 2022-11-18

**Authors:** Sina Salajegheh Tazerji, Roberto Nardini, Muhammad Safdar, Awad A. Shehata, Phelipe Magalhães Duarte

**Affiliations:** 1 Department of Clinical Science, Faculty of Veterinary Medicine, Science and Research Branch, Islamic Azad University, Tehran P.O. Box. 1477893855, Iran; 2 Young Researchers and Elites Club Science and Research Branch, Islamic Azad University; Tehran P.O. Box. 1477893855, Iran; 3 Istituto Zooprofilattico Sperimentale del Lazio e della Toscana “M. Aleandri”, 00178 Rome, Italy; 4 Department of Breeding and Genetics, Cholistan University of Veterinary & Animal Sciences, Bahawalpur 63100, Pakistan; 5 Avian and Rabbit Diseases Department, Faculty of Veterinary Medicine, University of Sadat City, Sadat City 32897, Egypt; 6 Research and Development Section, PerNaturam GmbH, 56290 Gödenroth, Germany; 7 Prophy-Institute for Applied Prophylaxis, 59159 Bönen, Germany; 8 Postgraduate Program in Animal Bioscience, Federal Rural University of Pernambuco (UFRPE), Recife, Pernambuco 52171-900, Brazil

**Keywords:** zoonoses, climatic changes, anthropic actions, emerging diseases, Hendra virus, rabies, hantavirus, leptospirosis, COVID-19, tuberculosis

## Abstract

Population growth and industrialization have led to a race for greater food and supply productivity. As a result, the occupation and population of forest areas, contact with wildlife and their respective parasites and vectors, the trafficking and consumption of wildlife, the pollution of water sources, and the accumulation of waste occur more frequently. Concurrently, the agricultural and livestock production for human consumption has accelerated, often in a disorderly way, leading to the deforestation of areas that are essential for the planet’s climatic and ecological balance. The effects of human actions on other ecosystems such as the marine ecosystem cause equally serious damage, such as the pollution of this habitat, and the reduction of the supply of fish and other animals, causing the coastal population to move to the continent. The sum of these factors leads to an increase in the demands such as housing, basic sanitation, and medical assistance, making these populations underserved and vulnerable to the effects of global warming and to the emergence of emerging and re-emerging diseases. In this article, we discuss the anthropic actions such as climate changes, urbanization, deforestation, the trafficking and eating of wild animals, as well as unsustainable agricultural intensification which are drivers for emerging and re-emerging of zoonotic pathogens such as viral (Ebola virus, hantaviruses, Hendravirus, Nipah virus, rabies, and severe acute respiratory syndrome coronavirus disease-2), bacterial (leptospirosis, Lyme borreliosis, and tuberculosis), parasitic (leishmaniasis) and fungal pathogens, which pose a substantial threat to the global community. Finally, we shed light on the urgent demand for the implementation of the One Health concept as a collaborative global approach to raise awareness and educate people about the science behind and the battle against zoonotic pathogens to mitigate the threat for both humans and animals.

## 1. Introduction

According to the World Organization for Animal Health (WAOH), 75% of the emerging diseases find their origin in domestic or wild animals, thus they are zoonotic, which prompts for a close collaboration between the animal and public health authorities [1]. The term emerging zoonosis is defined by the World Health Organization (WHO), the Food and Agriculture Organization of the United Nations (FAO), and WAOH as a newly recognized or evolved pathogen, which is a recent one or it has occurred previously, but which shows an increase in its incidence or expansion in the geographic area regarding the number of hosts or vectors [2]. The emergence of zoonotic diseases typically occur as consequences of several drivers such as: (i) Anthropogenic action such as urbanization, agricultural expansion, and deforestation, globalization, socio-economic development, agrochemical usage and the application of antimicrobial treatments as well as other behaviors (such as bush meat consumption, animal production and marketing, animal–human interfacing, and globalization); (ii) Environmental factors (such as temperature, drought, and wind); (iii) Biological drivers (such as genetic drift and reassortment) [3,4]. These factors have been proposed as direct or indirect contributors in the emergence and re-emergence of pathogens such as Ebola virus, Hendra virus, Middle East Respiratory Syndrome Coronavirus (MERS-CoV), Nipah virus (NiV), and the recently emerged severe acute respiratory syndrome coronavirus disease-2 (SARS-CoV-2) [5,6,7,8,9,10]. In this review, we will discuss the most relevant anthropogenic activities that are associated with the emergence and re-emergence of some zoonotic diseases.

## 2. Anthropogenic Actions

**Figure 1:**
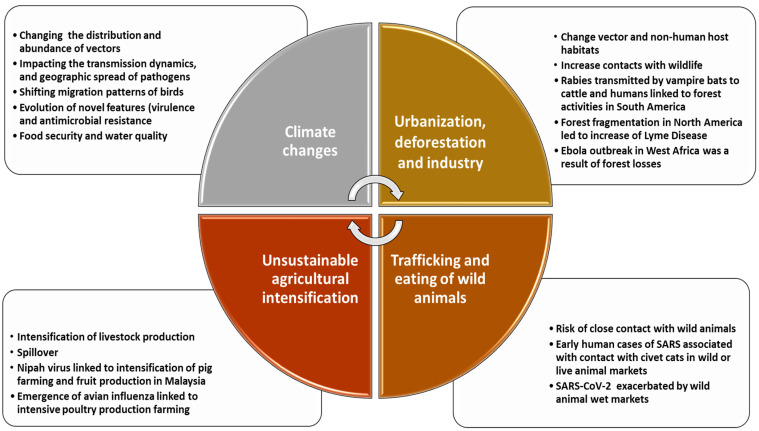
The main drivers of zoonotic outbreaks. Environmental imbalances such as climate changes, unsustainable agricultural intensification, trafficking, and consumption of wild animals as well as urbanization and deforestation are drivers for the emergence or re-emergence of diseases.

A summary of the potential impacts of anthropogenic actions such as climate changes, urbanization, deforestation, the trafficking and eating of wild animals, as well as the unsustainable agricultural intensification as the drivers for the emergence and re-emergence of pathogens are illustrated in Figure 1.

### 2.1. Climate Change

According to the Intergovernmental Panel on Climate Change (IPCC), climate change is a statistically significant variation in the average climate parameters (including its natural variability) [11,12]. It is defined by the United Nations Framework Convention on climate change as “a change of climate which is attributed directly or indirectly to human activity that alters the composition of the global atmosphere and which is in addition to natural climate variability observed over comparable time periods” [13]. Although the process of global warming can be occur by natural processes, the action of man, without a doubt, has been accelerating this process over recent decades. The loss of biodiversity, whether it is in terrestrial, aquatic and marine ecosystems, as well as the deterioration of the ecosystem’s services, result from anthropogenic interference, such as urban expansion, deforestation, and agriculture [14].

Indeed, the climate crisis has arrived, and it is accelerating faster than most scientists expected it to [15]. During the last 50 years, humanity’s ecological footprint has increased by nearly 190%, indicating that there is a growing unbalance in the human–environment relationship, which has been coupled with major environmental and social changes [16]. Climate change could drastically affect the human population. Migratory waves to more favorable environments and the interiorization of coastal populations to the continent may occur in the future. The global mean sea level, for example, has risen between 16 and 21 cm since 1900, and it has continued to rise at a rate of more than 3 mm per year over the past two decades [14]. A key activity that has accelerated climate change, and consequently, interfered with the dynamics of diseases is deforestation. The destruction of these natural habitats causes an increase in the amount of contact between wild animals and human beings, either by the human activity itself or by the adaptation of some species to the anthropic environment [17]. Another important foundation is the change in global temperatures. Several infectious agents and their vectors lack thermostatic mechanisms [18,19]. Therefore, the factors that affect the temperature can modify the geographic distribution of pathogens and their vectors [20]. Additionally, the presence of hemolytic bacteria in arctic environments demonstrates the risk that melting can introduce by bringing these bacteria in contact with human or wildlife animals, and this melting results from the increase in global temperatures, which is associated with the destruction or fusion of this habitat, ecosystem transition, and recolonization [21].

### 2.2. Deforestation

One of the most likely factors that explains the recent occurrence of new diseases is the expansion of the human population [22]. It is estimated that the world population will reach 10 billion by 2050. As big cities become overburdened, people tend to look for new spaces to live, moving into areas that were previously occupied by forests or other natural habitats. The population increase has also forced a greater production of food, which makes the natural areas a target for the occupation of the agricultural sector, use them for the production of food. According to [23], three interrelated world trends may be exacerbating the emerging zoonotic risks: income growth, urbanization, and globalization. The deforestation of these areas can lead to a decrease in biodiversity, generating several imbalances in the ecosystem. Consequently, deforestation can increase the occurrence of new cases of zoonotic diseases [24].

In this sense, the deforestation and burning of the Amazon Forest have fundamental roles in the degradation of the health of our planet [25]. In the first eight months of 2021, the deforestation rate in the Amazon was 8.2% higher than that which was recorded in the same period in the previous year, according to the National Institute for Space Research (INPE). Preliminary data from the TerraBrasilis platform show that from the beginning of 2022 to August, 11 million km² of forest were destroyed [26]. This situation is very worrying because, for example, 1 Km^2^ of deforested Amazon Forest could be equivalent to 27 new cases of malaria [27]. Due to the change in the rainfall regime, significant effects such as the outbreaks of infectious diseases which are transmitted by insect vectors and through contaminated water are expected to occur [28]. An example of this is the Amazon rainforest, which accounts for 11,000 km² of deforestation per year, thereby strongly impacting global warming, and affecting the regional climate of South America, with it causing changes in the transport of water vapor [29].

The destruction of forests is often conducted to open up new areas for agriculture, livestock, or mining. Human activity in forested areas puts humans in close contact with wildlife [30,31]. The association between the human activities in the Amazon rainforest, climate change, the changes in the vector dynamics, human migration, the genetic changes in pathogens and the precarious social and environmental conditions in many Latin American countries can give rise to the “perfect storm” for the emergence and resurgence of human infectious diseases in Brazil and in other Amazonian countries [32]. The number of diseases such as rabies, which are known to occur as a result of the deforestation and invasion of wild areas, tends to increase. In the Amazon Basin in 2004, 46 people died from this disease [33].

Brazil has a notable position in the agricultural sector as being one of the two largest grain producers in the world, with an estimated growth in grain production having occurred when it was compared to the 2020/21 season, with there being emphasis on soybeans and the total amount of corn [34]. The recent data indicate that three out of every four hectares of public lands that were deforested gave way to pasture for cattle ranching in the Amazon [35]. Another important biome has also suffered the impacts of anthropic actions. In the Brazilian Cerrado, which is home to 5% of the planet’s animals and plants, a third of its area (32.8%) was devastated for cattle and soy production between 2004 and 2017 [36].

Although deforestation is often intended to increase the food production, its consequences go against the grain of climate balance, a factor that is extremely sensitive and important for maintaining the productivity of vegetables and animal protein. In a study that was carried out by [37], in which they evaluated whether the expansion and intensification of the agriculture on the Amazon–Cerrado agricultural border were approaching a climatic limit for rainfed production systems, it was pointed out that future climate change could reduce the land area within the ideal climate space by more than 51% by 2030. It is essential that global public policies are implemented to protect the world’s main forests, and in addition to representing an important link with the planet’s climate scenario, they are essential for maintaining the balance between the pathogens and the natural hosts, and consequently, they are a key link for the emergence and re-emergence of diseases.

### 2.3. Trafficking and Consumption of Wild Animals

Food security is essential in preventing the emergence and re-emergence of diseases, and consequently, in ensuring human health. The current pandemic, the coronavirus disease 2019 (COVID-19) one, has had multiple impacts on food production, animals, and human health [38,39,40]. Poverty, which has been exacerbated by the pandemic, is a factor that must be considered, in the sense that hunger can lead to the consumption of wild animals [41]. Although there are few updated data on the consumption of animals of wild origin, it is known that they are part of the diet of several populations around the world. According to an FAO report entitled “The State of the World’s Biodiversity for Food and Agriculture”, in some communities in Asia, Africa and Latin America between 2004 and 2010, more than 53.5% of households were supplied with wild animals and plants [42].

In a study that was carried out in Brazil with the objective of verifying the demand and the potential of the commercialization of wild meats in the Municipality of Rio Branco, Acre/Brazil, 550 people were interviewed. Of those who were interviewed, 78% of them stated that they consume or have consumed wild animal meat [43]. In other studies, it is possible to verify the high diversity of the animals that are used in human food [44,45]. Within this aspect, it is important to highlight the conditions to which the communities are exposed. For example, in isolated regions, such as villages, hunting is the main source of food [46]. In the Amazon, the meat of game animals represents an essential part of their basic diet in several communities in the region [47].

China is one of the biggest consumers of wild animals for food and traditional Chinese medicine in the world [48]. Although the origin of SARS-CoV-2 is still under debate, the initial cases were associated with the Huanan South China Seafood Market [49]. According to Naguib et al. [50], live and wet markets serve as hubs where humans and different animal species are in close proximity to each other, but they are also crucial for the food supply in many countries. Live and wet markets have been linked to the emergence of different epidemic/pandemic diseases, including COVID-19 and different subtypes of influenza A viruses, and they are also an important source of foodborne pathogens. In addition to being potential sources of the transmission of several known microorganisms and parasites to humans, such as tuberculosis [51], leptospirosis [52], rabies [53], and brucellosis [54], these animals may contain several pathogens that have not yet been identified.

An additional concern in the consumption of these animals is antimicrobial resistance (AMR), such as Extended-spectrum β-lactamase and AmpC (ESBL/AmpC)-producing *Escherichia coli,* which was detected in wild boar, which is a topic of fundamental importance in One Health [55]. It is essential that food inspection and surveillance tools are strengthened to ensure food and health safety for humans and animals. However, it is also essential that this control comes from the citizens through public policies of the awareness of food, environmental and social education. Within this aspect, the trafficking of wild animals is a topic that must be discussed and approached more often. This trade, which is estimated to be worth between USD 7–23 billion a year, is the world’s fourth most lucrative trafficking industry after drugs, humans, and weapons [56].

In addition to the loss of species and the risk of extinction, the traffic of animals interferes with and attacks the ecosystems in which these animals live, unbalancing these habitats. Thus, the possibility of microorganisms adapting from these animals to humans arises. In addition, there is an illegal trade network of wild animals which are used for food supplies in many markets [48]. With this, the importance that public bodies play in ensuring this control is observable. China, for example, in the face of COVID-19, decided to ban the trade and consumption of wild terrestrial animals. The ban has implications that extend beyond safeguarding human health to also help to combat illegal trade and protect endangered species [57]. Wildlife trade controls are very limited because of the bias for the utilization of wildlife as a natural resource that is to be exploited by government agencies. The key to public awareness publicity and education is to provide more information on the negative impacts of wildlife consumption and knowledge about their protection [58].

## 3. Human–Host–Environment Interaction

**Figure 2:**
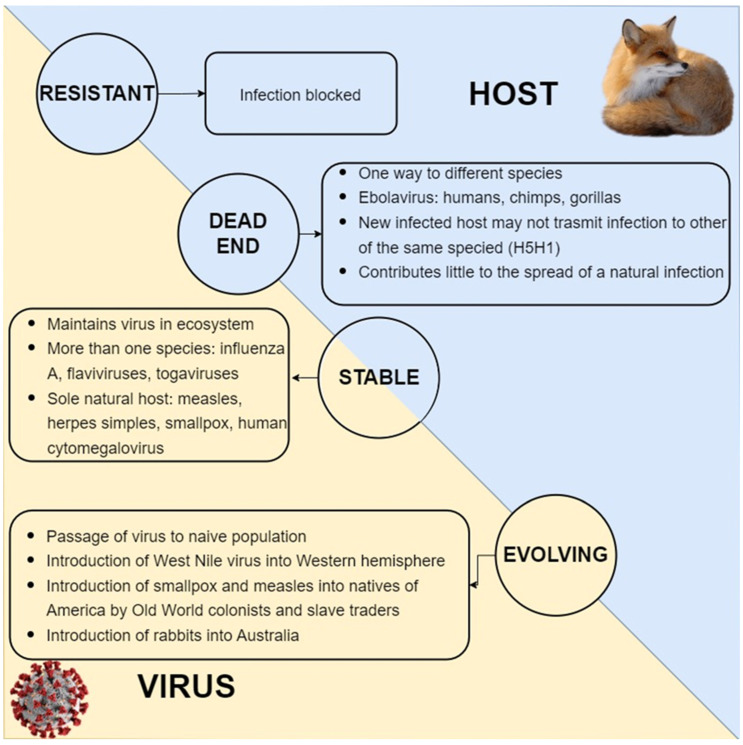
Host–virus interactions. The host–virus interactions are hypothesized to be a stable interaction (maintains the virus in the ecosystem), which includes an evolving interaction (passage of virus to the naive population in the same or other host species), a dead-end interaction (one way to different species), and a resistant host interaction (completely blocks infection).

The interaction between the host, the host microbiome, the pathogen, and the environment is called a four-way interaction, and it is complex, and it explains the emergence of pathogens and predicts the epidemic risks due to anthropogenic actions (Figure 2) [59].

Anthropogenic actions, for example, drive the increasing rate of wildlife-human contact and the human-driven introductions of pathogens by providing conditions that promote our interaction with wild animal populations due to fundamental changes in the environment [60,61,62,63,64,65,66]. These impacts are not restricted to the emergence of zoonotic viruses, however, anthropogenic pollutants have been linked to several chronic diseases such as Parkinson’s disease and diabetes [67,68,69,70]. Additionally, it was proposed that bacteria may possess cross-tolerance or cross-resistance properties for herbicides glyphosate (N-phosphonomethylglycine), leading to the emergence of ESBL-producing *Enterobacteriaceae* [71]. The overuse or misuse of antibiotics can lead to the emergence of antimicrobial resistance (AMR) [72,73,74], however, the emergence of carbapenem resistance is increasingly being reported, and therefore, it presents a significant public health threat in Africa, although carbapenems are generally unavailable in African hospitals [75]. Therefore, this set of environmental changes favors the interaction of pathogen agents with their vector, and with wild and domestic hosts, in addition to humans [76]. Consequently, there can be serious implications for environmental dynamics, such as the disappearance of species that serve as natural hosts for potential pathogens. As a result, these agents could spill over to other hosts, including humans.

## 4. Selected Emerging and Re-Emerging Viral Pathogens

### 4.1. Ebola Virus

Ebola virus, belongs to family *Filoviridae*, and it is an enveloped, single-stranded, negative-sense RNA virus of approximately 19 kb [77]. The *Filoviridae* family is divided into three genera: *Ebolavirus*, *Marburgvirus*, and *Cuevavirus*. The genus *Ebolavirus* contains five distinct species, namely, Zaire ebolavirus, Sudan ebolavirus, Taï forest ebolavirus, Bundibugyo ebolavirus, and Reston ebolavirus, which is represented by EBOV, Sudan virus (SUDV), Taï forest virus, Bundibugyo virus (BDBV), and Reston virus, respectively [78]. In the most recent decade, EBOV, SUDV, and BDBV have produced Ebola virus disease (EVD) epidemics in Central and West Africa with increased frequency, and the case fatality rates range from 30% to 90% [79]. Bombali virus (BOMV), which is a novel ebolavirus belonging to the proposed new species Bombali ebolavirus, was recently detected in bats in Sierra Leone and Kenya [80,81]. Mengla virus (MLAV) was also discovered in fruit bats in China. Olivero et al. [82] investigated the effect of anthropogenic actions on the emergence of EVD. It was proposed that there is a significant link between forest degradation and its fragmentation and human EVD outbreaks. Deforestation has the potential to alter the composition, abundance, behavior, and perhaps exposure of reservoir species. As a result, the interaction between the infected animals and humans is increased [82].

### 4.2. Hantaviruses

Hantaviruses, belonging to *Bunyaviridae* family, are an RNA single-stranded negative-polarity virus that can cause two types of infection in humans: hemorrhagic fever with renal syndrome (HFRS) and hantavirus cardiopulmonary syndrome (HCPS). Twenty-eight hantaviruses have been identified so far. The reservoirs of the virus include rodents, insectivore hosts and bats, which can infect human mostly by the inhalation of contaminated aerosolized rodent excreta [83].

The infection is correlated to human–rodent interactions, and so in developed countries, some professional people are more exposed to the risk of contracting the disease (forest workers, pet rats owner, laboratory personnel trapping workers, hunters) [84,85,86,87], meanwhile, in rural or developing countries, the risk is also widespread among the general population [88,89]. Several serological studies have been conducted in many countries of the world, revealing the variable prevalence of it from 6% to 36% [90,91,92,93,94,95]. The concern for these viruses is high, so much so [96] that it is hypothesized whether they could be responsible for the next pandemic. The influence of the landscape and other environmental factors were analyzed in [97], in which climatic variables, land use variables, vegetation indices, soil variable and human distribution were identified as factors affecting the risk of hantaviruses. The same factors are all influenced by anthropic actions.

### 4.3. Hendravirus

Hendra is closely related to the *Henipavirus* genus, with it having around 78% nucleocapsid (N) gene sequence homology with NiV [98]. Hendra viruses were first described in Australia in 1995. They cause severe infections in horses, and under experimental conditions, it infects cats and guinea pigs [99]. In humans, it causes severe encephalitis (inflammation of the brain), which is accompanied by respiratory symptoms. Similar to NiV, the reservoir for Hendravirus is the fruit bat of the genus *Pteropus*, which are found in a wide swath from south and southeast Asia to northern and eastern Australia, as well as in Madagascar and some islands of the western Pacific. The expansion of the human populations into the wildlife habitats appears to be the primary driver of the introduction of Hendra virus [64].

### 4.4. Nipah Virus

**Figure 3:**
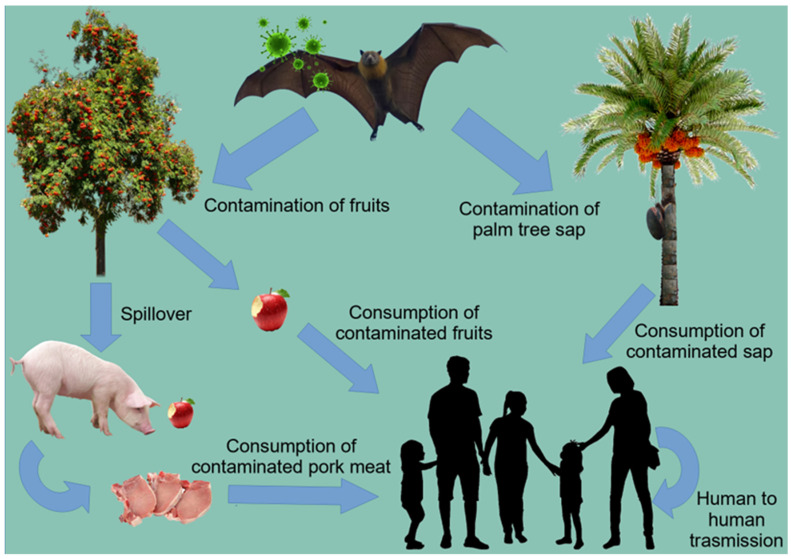
Transmission of Nipah virus (NiV), adapted after [108]. Fruit bats are the natural reservoir for NiV Transmission occurs via contact with infected animals, ingestion of contaminated fruits, consumption of contaminated pork, or human-to human transmission.

The NiV belongs to the family *Paramyxoviridae,* and it is an enveloped pleomorphic virus of the genus *Henipavirus* [100]. The genome of the virus is represented by a non-segmented negative-sense single-stranded RNA, which encodes six structural proteins, namely, N, phosphoprotein (P), matrix protein (M), fusion protein (F), glycoprotein, and RNA polymerase [101]. While the G protein mediates binding with the host cellular Ephrin-B2 and -B3 receptors, the F protein induces the viral–cell membrane fusion that facilitates the virus’ entry [102,103]. The natural reservoir for the NiV is the fruit bat of the genus *Pteropus* (flying foxes), which are endemic in tropical and subtropical regions of Asia, East Africa, Australian continents, and some oceanic islands [104,105]. The route of transmission occurs via contact with the excretions or secretions of infected animals, the ingestion of fruit that is contaminated with NiV, or close contact with infected human bodily fluids [106,107] (Figure 3).

The emergence and transmission of NiV could be attributed to several anthropogenic factors: (i) Density population: The NiV outbreaks were reported in regions with the densest populations in the world such as Kerala (Bangladesh) and in the south Indian states [109,110]. The high population density mediates a high rate of interaction between the individuals and the environments. Additionally, the co-existence of farm animals in regions of dense human inhabitation generates a high risk of virus spillover [108]. (ii) Deforestation: Due to the loss of bat habitats, climate changes and deforestation has enforced bats to resort to fruiting trees which has led to the spillover of the virus to the pigs or directly to humans via the consumption of bat-bitten fruits [106]. An NiV outbreak was reported in Malaysia (1998–1999) and in Kerala (2016) following the drought and deforestation due to El Nino [111,112]. (iii) Reservoir Distribution (demography): bats have been driven to remain close to human communities in metropolitan areas across the world due to habitat loss [113,114]. Further, bats are the reservoir of NiV, which uses pigs as an intermediate host. The NiV virus may also be transmitted directly from bats by the ingestion of contaminated date palms. The hunting of bats for human consumption should be also considered [115,116]. (iv) Socio-economic scenarios: In Malaysia, the NiV outbreak originated in pigs, the main source of income for farmers [117]. In Bangladesh, date palm sap was the main source for the NiV infection [118]. Transportation, tourism, and high proportions of health care units in the West Bengal and Kerala states account for the high rate of nosocomial NiV incidences [108].

### 4.5. Rabies

Rabies is an ancient, underreported, and progressive neurological zoonotic disease with nearly a 100% mortality rate [119]. It is caused by a single-stranded RNA virus that belongs to the Lyssavirus genus of the *Rhabdoviridae* family [120]. Although rabies can be prevented by vaccines, about 59,000 people die from rabies each year, globally [119]. Rabies is endemic in many countries, except for Australia and Antarctica and rabies, and it is more common in developing African and Asian countries due to various factors including rapid urbanization, a high volume of waste, and a lack of vaccination facilities and proper hygiene [121,122]. Rabies circulates through the urban cycle, including interactions between domesticated and stray dogs, and through the sylvatic cycle with interactions occurring among wild animals such as foxes, wolves, jackals, mongoose, raccoons, skunks, and bats. These two cycles are interrelated and sometimes overlapped [119]. In developing countries and developed countries, dogs and wildlife, respectively, are the main causes of rabies transmission [123]. Rabies is believed to affect all mammals, however, only some of them are reservoirs of the virus [124]. Domestic animals (cats, cattle, and dogs) account for less than 10% of the reported rabies cases [125,126]. Rabies is often transmitted by the saliva and bite of an infected host. Moreover, this infection can also occur through scratches, aerosols, organ transplants, and body fluids such as tears [119,127]. Anxiety, bewilderment, hallucination, and hydrophobia are some of the symptoms of this disease [128]. A variety of social and environmental factors have been shown to play a role in emerging and re-emerging zoonoses such as rabies. Urbanization, deforestation, and waste accumulation are the most significant among them [129,130].

#### 4.5.1. Rabies and Urbanization

Today, 54% of the world’s population reside in cities. Urbanization can facilitate the spread of rabies in cities and complicate its control because of its social and spatial aspects [131]. Urbanization causes population displacement and migration by providing employment, higher salaries, and better health care services [130]. Rural migrants, due to their connection to rural wildlife and the animals that may bring to the city, increase the risk of rabies transmission [132]. Bat bites in humans were originally documented in rural regions, but they now also occur in urban areas [133]. International travelers are exposed to intentional or unintentional contact with animals that coexist with humans, and as a result, travelers are predicted to be exposed to the rabies virus at a rate of 0.4 per 1000 for every month that they spend abroad [134,135].

Illegal imports, natural migration, and translocation (both purposeful and involuntary) may facilitate the entry of rabies viruses into virus-free areas [136,137]. Over the last decade, the interest in companion animal travel programs to improve the outcomes for dogs and cats in animal shelters has grown significantly in North America and Europe [136]. Garbage trucks and other vehicles can accidentally transport raccoons and other wildlife species that scavenge among human waste [138].

Some urban structures, such as water canals and roads, increase the spread of rabies [131,133,139]. Garbage that is dumped in water canals increases the density of the dogs around them. They also create barriers for pedestrians and limit the access to rabies vaccination centers [131]. Bats use mines, tunnels, wells, culverts, and abandoned houses to reside in, which helps to enhance the bat population [133,140].

#### 4.5.2. Rabies and Garbage Accumulation

The rising population of cities has a considerable influence on the amount of household garbage that is produced. Urban populations create two to three times the amount of municipal garbage (measured in kilograms per capita per day) as rural residents do. Unfortunately, most of the local governments and municipalities are not able to manage and remove this amount of waste, which leads to the accumulation of waste in residential areas and open dumping grounds [141]. Only around 25% of the total amount of garbage that is created in Europe is placed in landfills, with the rest of the waste being composted, recycled, or burnt, whereas Asian rabies-endemic nations dump over 85% of their generated waste. This number reaches almost 97% in African countries [121]. Open garbage dumps are a public health obstacle in the community and lead to the proliferation of stray dogs [142,143]. Accumulated waste increases the number of possible vectors for rabies by providing food and habitats for animals such as stray dogs and omnivorous raccoons [130,142,144]. Dogs that obtain their food from garbage that is left by bakeries and abattoirs do not recognize that humans are the food suppliers, and they are more likely to attack them [142]. The aggression of hungry dogs competing for food endangers the residents and increases the risk of rabies transmission to people, dramatically [145]. Proper waste management is necessary to minimize the population of potential rabies carriers.

#### 4.5.3. Rabies and Pets

Growing urbanization is causing an increase in the number of conventional pets such as cats and dogs in households, which may increase the danger of contracting rabies [146]. Millions of cats are kept as pets, with 34% of households keeping cats in the United States, 26% of them keeping cats in the European Union, and 27% of them keeping cats in Australia [147,148]. Additionally, in recent years, the population of domestic dogs in European countries, the United States, and India have increased by 6–7.7%, 15.29%, and 65%, respectively [121,149,150]. Bites by an infected pet dog (*Canis familiaris*) are a major cause of human rabies [151]. Many pet owners live in multi-unit apartments, which increases the amount of contact between the people and the pets in the surrounding environment, such as in playgrounds and recreational areas. In addition, approximately 14–62% of them permitting their pets to have entry into their bedrooms [129,146]. In urban areas, some domestic dogs and cats are poorly monitored or roam freely, so they are more likely to come into contact with wildlife and other rabid animals [147]. Pet cats can hunt wildlife such as bats, and humans are more likely to get rabies from the bites of these cats [147,152,153]. Taking proper care of pets, compulsory vaccinations, the monitoring of their habitat, and having training on wildlife and pet risk factors are some of the factors that can help reduce the risk of rabies [130].

#### 4.5.4. Rabies and Deforestation

Human population growth, urban development, increasing land productivity, mining, dams, and deforestation are some of the factors that cause wildlife to overflow into human environments [154]. In 1990, there were 4129 million hectares of forest on the planet, whereas in 2015, this had decreased to 3999 million ha [155]. By converting many of these forests into agricultural or urban development land, natural wildlife habitats have been reduced, and the number of wildlife interactions with humans and domestic animals is enhanced [122,130,154]. Bat colonies are located in urban areas that are close to human homes, which increases the risk of sustaining injuries through bat bites [122,152]. Bats have made it difficult to eradicate rabies due to their aerial lifestyle and the problems in developing and prescribing vaccines for them [156]. In Taiwan, the outbreak of rabies in ferret badgers has endangered the long-term stability of rabies vaccination among dogs [157]. In modified anthropogenic areas, raccoons have a superb relationship with the cities, and they made little use of forest cover because of the human resources and the shelter that they offer from larger predators [158].

#### 4.5.5. Rabies and Food Supply

Following the increase in the population and the need for food, the number of livestock animals and dogs has increased [122,130]. Increasing the number and habitats of different livestock animals in the same environment creates a dynamic microenvironment that aids interspecies transmission [130]. Additionally, in Latin America, where livestock production is a primary source of food, there has been an increase in the risk of hematophagous bat attacks in recent years [159]. Dog slaughtering is also one of the means of transmission in many countries because dog meat is a popular food in places such as China, South Korea, and Ghana. Although dog meat does not cause the disease, the risk of transmission increases during correlated activities. Moreover, most butchers do not know enough about rabies, and the slaughterhouse environment is unsanitary [122].

### 4.6. SARS-CoV-2

COVID-19, which is caused by SARS-CoV-2, is a new pandemic that emerged in December 2019 in Wuhan, Hubei Province of China [160,161]. To date (6 October 2022), about 625,079,727 confirmed cases and 6,555,942 deaths have been reported worldwide. Although several recommended preventive measures such as vaccinations, lockdowns, test, trace, and isolation measures, wearing masks, social distancing, and the frequent washing of hands were implemented to control this pandemic, several ongoing challenges are still being faced [162].

Several anthropogenic factors (human-related factors) have influenced the transmission and spread of SARS-CoV-2: (i) The COVID-19 pandemic was exacerbated by wild animal wet markets [163]. It is widely argued that people became infected with SARS-CoV-2 through the interaction with wild animals at the Huanan seafood wholesale market [164]. (ii) Globalization (international travel and trade). The outbreaks occurred at an extraordinary frequency and speed as a result of the globalized environment of interconnected trade, travel, and migrations, and the infection does not function along geopolitical borders. Travel limitations can only lead to delays in the epidemic peaks that last from a few days to a few weeks. Therefore, the early detection of the outbreaks, and performing hygienic measures, self-isolation, and household quarantine were more successful at limiting the pandemic than travel limitations would have been [165]. Additionally, the molecular epidemiology of SARS-CoV-2 could explain the critical role of air travel in the global spread of SARS-CoV-2 [166,167,168]. (iii) The demographic changes in the population size and density. Urbanization affects the dynamics that lead to persistent outbreaks in more populous, denser urban region [169].

## 5. Selected Bacterial Diseases

### 5.1. Leptospirosis

Leptospirosis is a common bacterial zoonotic disease. This disease is prolific, worldwide, because it is caused by a wide range of host mammals [170,171,172]. In addition, the studies show that birds, amphibians, reptiles, and fish also carry the causative agent of this disease [173]. It is one of the most prominent causes of morbidity and mortality, especially in tropical zones [171]. Leptospirosis is caused by *Leptospira spp*, which are helical and highly motile spirochetes [174]. Leptospirosis is transmitted directly from one host to another or indirectly through soil, contaminated water, and infected animal urine. This microorganism enters the body through the skin, mucous membranes of the mouth, and conjunctiva, and it then causes the disease [175].

Leptospirosis causes 60,000 deaths, worldwide, each year [171]. Over the past few decades, it has been a severely neglected and underestimated threat. Many studies show that leptospirosis is re-emerging, and it is becoming a public health problem, worldwide, with significant increases in its incidence and there being multiple outbreaks [176]. Recently, the disease has become widespread in Nicaragua, Brazil, India, Southeast Asia, the United States, and in several other countries [177,178,179,180,181,182,183,184]. Despite this, there are a few reports of leptospirosis in South and Southeast Asia, especially in the densely populated countries such as India and Indonesia, because the monitoring of it is very poor [171]. Various factors are effective in the occurrence of this disease. One of the most important of these factors is the rise in urbanization. Global warming, severe climate change events such as floods, increasing poverty and marginalization, urban sprawl, and the destruction of wildlife habitats, and increasing contact with rodents and domestic animals such as dogs and cats are all due to a rise in urbanization.

#### 5.1.1. Leptospirosis and Urbanization

Urbanization has been occurring for more than 250 years, but only in the 21st century has it become a global feature, especially in the poorer parts of Asia and Africa [185]. Excessive urban population growth causes the cities to expand more rapidly than the number of jobs and houses can. Under these conditions, urban slum communities expand with poor sanitation infrastructure, the presence of vermin, a lack of waste disposal facilities, and poor water quality [186]. Rodents are the most important reservoir of Leptospira [187]. Rodents such as wild rats grow in urban and domestic environments, which leads to frequent instances of human exposure to them [188]. Due to there being little knowledge of rat ecology, controlling rats is largely impossible [189]. In a study that was conducted in Baltimore, USA, Leptospira were isolated from 95% of the trapped mice [190]. The prevalence of Leptospira in the populations of urban rodent species in Switzerland has also been reported to be between 10 and 20% [191].

#### 5.1.2. Leptospirosis and Extreme Weather Events

Extensive urbanization increases greenhouse gas emissions, global warming, and the amount of heavy rainfall [192]. The expansion of the cities can cause floods by destroying the main river routes [176,193]. Additionally, in recent years, there has been an increasing amount of rainfall, and storms and floods occur more intensely, which may lead to an increase in the prevalence of leptospirosis [194,195]. In Brazil, it was estimated that for every millimeter of daily rainfall per month, the number of leptospirosis cases increased by 0.55% when it was compared to the average of that period [196]. Global warming can also be a factor in increasing the likelihood of Leptospira surviving in the environment [197].

#### 5.1.3. Leptospirosis and Socio-Economic Phenomena

Changes in the economic statuses following urbanization, such as impoverishment and homelessness, increase the incidence of leptospirosis. It is also very worrying that rodents and domestic animals are increasingly exposed to the living environment of homeless people or people living in slums or uninhabited neighborhoods of cities wherein Leptospira is transmitted to them [190,198,199]. Leptospirosis is considered to be an important disease in the poor parts of Europe [200]. In industrialized and developing countries, the migration from rural to urban areas has caused urban epidemics [201]. The prevalence of Leptospira is at 16% among Baltimore residents in the US, and it is at 30 percent among the children in Detroit neighborhoods [202,203].

#### 5.1.4. Leptospirosis and Pets

Cats and dogs have a significant relationship with humans, and they are popular pets around the world. In the US, 40.1% of households own a dog and 26.5% of them own a cat, while in the EU, 26% of households own a cat and 24% of them own a dog [204,205]. Recently, some epidemiological studies have reported the risk of the transmission of Leptospira to humans from pets. Dogs are known to be a potential reservoir of Leptospira. However, they were commonly infected with *Leptospira canicola* and *icterohaemorrhagiae*. These two serovars are commonly used in polyvalent vaccines in dogs. Vaccines have effectively prevented the transmission of these two serovars to humans. Recently, some studies have shown the development of leptospirosis from serovars such as *Leptospira autumnalis* or *pomona*, which have previously been rarely found in dogs [206,207]. The antigens of these new serovars are not yet present in the vaccines, so it is possible to cause and transmit the disease from "vaccinated" dogs [176]. The transmission of leptospirosis from dogs has also been shown to be one of the most important causes of human leptospirosis in the last two decades in Russia [208].

#### 5.1.5. Leptospirosis and Wildlife Animals

The intrusion of wildlife species living in the suburbs increases the potential risk of the Leptospira transmission from animals to humans. Urban development, increasing the population densities in the cities, and occupying non-residential areas allow them to have easy access to food. Wild boars, foxes, deer, martinis, skunks, and raccoons can be seen frequently not only in the suburbs, but also sometimes in old urban neighborhoods. In a study in Berlin, *Leptospira* was isolated from 18% of the wild boars in the suburbs [209].

### 5.2. Lyme-Borreliosis

Lyme-Borreliosis, which is also known as Lyme disease, was initially discovered in Lyme, Connecticut (USA) in 1975. Later on, the disease has been reported in North Ameri-ca, Europe, South Korea, and Asia [210]. Lyme disease, which is a tick-borne disease, is caused by several bacterial species that cause clinical manifestations in the skin (Erythe-ma migrans). However, *B. garinii* and *B. bavariensis* are associated with neurological manifestations [211]. *B. afzelii* can develop acrodermatitis chronica atrophicans, while *B. burgdorferi sensu stricto* is associated with Lyme-Arthritis [212,213,214].

#### 5.2.1. Borrelia spp. and Geographical Distribution

*B. burgdorferi sensu stricto* is known to cause Lyme disease in North America and Eu-rope [212,215]. In 2011, it was isolated from wild rodents in South Korea and from human samples in Taiwan [216,217]. In 2016, a new pathogenic *Borrelia burgdorferi sensu lato* genospecies (*Borrelia mayonii*) was reported in the upper midwestern USA [218]. In Europe, at least five Borrelia spp. (*B. afzelii, B. garinii, B. burgdorferi sensu stricto, B. spielmanii*, and *B. bavariensis*) have been identified, in which *B. afzelii* and *B. garinii* are the predominant species. In Asia, all of the human pathogenic species except *B. burgdorferi sensu stricto* and *B. mayonii* have been identified; *B. garinii* is the predominant species. *Borrelia* spp. are known to infect numerous animal species including small mammals, lizards, and birds [219,220].

The tick, *Ixodes* spp, is the vector of Lyme disease, and it transmits the *Borrelia* spp. be-tween the different hosts. Tick is the only natural agent that is known to cause infections in humans [212,221]. The worldwide geographic distribution of borreliosis correlates to the concurrent presence of both the reservoir and ticks. In the northeastern and midwestern United States, *I. scapularis* (black-legged tick) is the predominant vector, whereas, in the western states [222], *I. pacificus* (western black-legged tick) is the main vector. In Asia and Europe, *I. persulcatus* (taiga tick) and *I. ricinus* (European sheep tick) are the main vectors for Lyme disease, respectively [223,224].

#### 5.2.2. Impacts of Climatic Changes and Anthropogenic Activities on Lyme Disease

Deciduous and mixed forests, pastures, and urban parks are the favored habitats for ticks [225]. These habitats ensure the optimal micro-environmental conditions such as humidity and temperature, particularly during the juvenile stages, which are most vulnerable to water loss [226,227]. Additionally, these habitats are populated by rodents, which serve as tick hosts, which are necessary for the tick life cycle [228]. Ticks are known to be highly dependent on the climate patterns, and their seasonal activity varies significantly depending on the thermal conditions [229,230,231,232,233]. The differences in the *I. ricinus* activity between the regions in Europe are mainly associated with differences in the thermal conditions and ecological habitat types, thus, 98% of the two-year life cycle takes place inside the host. Climatic changes play a vital role in the emergence and re-emergence of this disease through its impact on the vector distribution and activities [226,234].

Indeed, several anthropogenic activities impact the epidemiology of Lyme disease. In North America, most (>90%) of the borreliosis cases were reported in the northeast and mid-Atlantic region and the north-central region [235,236,237]. These regions have been subjected to a substantial expansion, highlighting the role of anthropogenic activities in the epidemiological distribution of Lyme disease [237]. In the U.S., James et al. (2013) found that Lyme disease increased as a result of the changes to land use and a sharp rise in the deer population which in turn increased the risk of exposure to ticks carrying *Borrelia* spp [216].

Brownstein and others predicted the influence of climate change on the epidemiology of Lyme disease and the likely public implications of it in North America [238]. The authors estimated a significant spread of *I. scapularis* northward into Canada by the 2080s with a 213% increase in the appropriate habitats. The authors suggested also that climate change will cause the vector to recede from the southern United States and move into the middle of the US. Collectively, studying the environmental parameters that are associated with tick abundance and the prevalence of the disease may be helpful in reducing risk and predicting the future distribution of borreliosis in the face of climate change.

### 5.3. Tuberculosis

Tuberculosis, which is caused by *Mycobacterium tuberculosis*, continues to be a global public health problem worldwide. According to the WHO report in 2022, 10.6 million tuberculosis cases were diagnosed worldwide in 2021 [239]. Additionally, multidrug-resistant tuberculosis still poses a threat to the public health.

McIver et al. investigated the indirect drivers of tuberculosis transmission in the Pacific atoll countries. The authors have summarized these drivers in three main risk factors “Triple Whammy” which are: (i) Socioeconomic (poverty, overall population density, and household-level overcrowding), and (ii) Smoking and non-communicable diseases (diabetes mellitus and malnutrition, and (iii) Climatic changes (extreme weather events and sea levels), highlighting the bidirectional relationship between tuberculosis and the environment [240,241,242,243].

The impact of climatic changes on tuberculosis transmission can be explained by it having numerous pathways, for instance: (i) Climatic changes have an influence on food security and nutrition through erratic rainfall patterns, extreme weather events, high temperatures, a reduction in the arable land due to saltwater infiltration, and by decreasing the crop production. In many high-tuberculosis-burden countries such as India, malnutrition is the biggest risk factor for tuberculosis [241]. (ii) Extreme weather may also force population displacement into cramped conditions, increasing the risk of tuberculosis transmission [243]. Collectively, predicting and limiting the impact of climatic changes on food security and water quality should be taken into consideration to eradicate tuberculosis in countries with a high incidence of the disease.

## 6. Selected Parasitic Diseases

### Leishmaniasis

Leishmaniasis is an anthropozoonotic parasitic that is caused by protozoa of the *Trypanosomatidae* family and *Leishmania* genus, with the species *L. infantum* being one of the main etiological agents of canine visceral leishmaniasis (CVL) and its human variant (HVL) [244]. The protozoan is transmitted through blood, and this is carried out by female sand fly vectors belonging to the genera *Lutzomyia* in the New World and *Phlebotomus* in the Old World [245]. The disease is considered to be one of the most neglected ones in the world, with there being a higher prevalence in populations with socioeconomic and food vulnerability [246]. Approximately 95% of the annual cases occur in just 10 countries: Bangladesh, Brazil, China, Ethiopia, India, Kenya, Nepal, Somalia, South Sudan, and Su-dan [247]. The main reservoirs of the disease in the urban cycle are infected dogs, especial-ly asymptomatic ones [248]. Humans are accidental hosts and do not seem to have an important role in maintaining the parasites in nature [249].

The occurrence of the cases of leishmaniasis is related to human actions, such as the urbanization and occupation of areas in a disorderly manner, resulting in environmental imbalances [250]. In this way, the deforestation and the occupation of these areas promotes the adaptation of sandflies to anthropized areas [251,252]. Additionally, even though environmental degradation can negatively affect the abundance and diversity of sand fly populations, many species end up successfully adapting to the degraded habitats [253]. It should be noted that the occurrence of cases among animals precedes the cases among humans [254]. Since the dog is the main reservoir in the urban cycle, practicing responsible ownership and the abandonment of animals should be discussed since transmission can occur transplacentally, through colostrum ingestion, and venereally, which is a problem when one is referring to stray dogs [255,256,257,258].

Climate change may facilitate zoonotic spillover through the modification of the environments and ecosystems, and with that, by altering the habitat of many animals along with their parasites and pathogens [259]. The effects of climate change can modify the distribution of leishmaniasis in three ways: directly, through the effect of temperature on the parasite and on the development and vector competence; indirectly, by the effect of temperature and other environmental variables on the range and abundance of sandfly species that act as vectors; indirectly, through socioeconomic changes that quantitatively affect the amount of human contact with transmission cycles [260]. In addition, changes in the global climate can lead to food shortages and famine, generating an increase in movement of populations and migratory waves, and consequently, allowing both the introduction of Leishmania into Leishmania-free areas, as well as the insertion of susceptible individuals into endemic areas [261]. 

## 7. Fungal Diseases

The prevalence of fungal diseases has increased alongside the rise of immunosuppressive diseases in human and animal populations. Although new antifungal drugs have been recently developed, the prevalence of fungal infections has continued to rise. As a result, the rate of drug resistance to these medications has also increased greatly, thereby posing serious health issues [262].

The potential impacts of anthropogenic activities and climatic changes on fungal diseases can be summarized as follows: (i) The emergence of new human pathogenic fungal species such as *Candida auris* [263]. This *yeast* was initially isolated in 2009 in Japan, and since then, it has spread globally [264,265]. The mechanism for the emergence of this *yeast* has been explained as the evolution of a “novel” human fungal pathogen in response to climatic change, or as a consequence of anthropogenic activities such as the expansion of farming and aquaculture as well as the use of fungicides [265,266]. (ii) By impacting the geographical distribution of fungal pathogens. As a result of climate change, pathogenic fungi or their vectors may spread, geographically, more widely, causing the emergence of diseases in regions where they had not previously been noted. Floods, storms, and hurricanes can disseminate and aerosolize fungi or deposit them into traumatic wounds, leading to infections by previously unusual or unknown fungal species [263,267]. Global climate change is also contributing to the geographical spread of pathogenic fungi, including dermatophytes, leading to higher numbers of dermatophytosis [268]. (iii) The evolution of being thermotolerant. The ability of the vast majority of fungal species to multiply at high temperatures restricts the colonization and infection of mammals. However, in response to an environmental stress such as global warming, the fungi may evolve to become thermotolerant, which could increase the number of pathogens [267,269]. Gadre et al. reported that persistently high temperatures lead to the expansion of the geographic ranges of the dimorphic fungi *Coccidioides, Blastomyces, Histoplasma*, and *Sporothrix* [270]. (iv) Environmental stresses may also promote the evolution of novel features such as virulence and antifungal resistance of fungal pathogens, including those that are traditionally considered to be human commensals such as Candida albicans [271,272].

## 8. Recommendations

Mitigating the effect of anthropic action on the spread of emerging and re-emerging diseases is a very complex matter that only a One Health approach could solve. The control of ongoing and future pandemics should involve international cooperation from governments, pharmaceutical companies, diagnosticians, epidemiologists, public health specialists, vaccinologists, and medical and veterinary clinicians. In order to implement the One Health strategy, the following measures are recommended: (i) Hiring professionals with the necessary training; (ii) Performing the rapid and accurate diagnosis and treatment of infected individuals and animals; (iii) Developing and providing vaccines for virus control in humans; (iv) enhancing the hygienic measures; (v) Employing veterinary expertise; (vi) The monitoring of wildlife for the identification and characterization of potential reservoirs and the monitoring of people who come into contact with wildlife to identify the risk factors in human behaviors and living conditions [160,161,273]. Regular epidemiological studies in regions, countries, and around the world can mitigate the risk and help to control and prevent zoonotic pathogens [274]. Therefore, it is crucial to identify the risk factors in detail to take intervention measures to control the pandemic [275].

Nonetheless, the scientific community, alone, apart from improving awareness of the risks to human health through the publication of reliable data, can contribute little to the change of perspective. Political actions at all levels, international, national, and local, are the only actions that are capable of reversing the real attitudes. The first big issue that is always overlooked, for ethic, economic, and religious reasons, is the increase in human population, which is constantly rising, and that should be addressed by introducing control measures. The second one is the economic gap between the nations, which has forced some populations to make choices that have the negative effects on the environment, and these are reinforced by some legal procedures such as selling fish or carbon stocks to foreign nations. The third one is to adopt a non-homogeneity stance on the political management of natural resources and the protection of the natural reserves.

Raising awareness and educating people about the drivers of emerging and re-emerging pathogens could also reduce the risk of infection among people. This included and not limited to: (i) Mitigating the risk of vectors-to-human transmission by limiting the vector access to food products, protecting animals and their feed from bats where applicable, as well as practicing hygienic measures, (ii) Mitigating the risk of animal-to-human transmission by reducing the amount of contact with wild animals, wearing gloves and other protective clothing while handling sick animals or their tissues and during slaughtering and culling measures, and (iii) Mitigating the risk of human-to-human transmission, through avoiding contact with infected/sick persons, and practicing regular hand washing.

## 9. Conclusions

Anthropic actions pose a determinant role in emerging and re-emerging diseases, and future pandemics could be worse than the past and ongoing pandemics have been/are because we are forcing nature to its limits by destroying the incredibly diverse ecosystems which will eventually remove the natural buffers and expand the interface between wildlife and people where pathogens emerge/re-emerge. Therefore, the multidisciplinary One Health efforts must be adopted and implemented, worldwide.
